# Chemical State of Potassium on the Surface of Iron Oxides: Effects of Potassium Precursor Concentration and Calcination Temperature

**DOI:** 10.3390/ma15207378

**Published:** 2022-10-21

**Authors:** Md. Ariful Hoque, Marcelo I. Guzman, John P. Selegue, Muthu Kumaran Gnanamani

**Affiliations:** 1 Department of Chemistry, University of Kentucky, Lexington, KY 40506, USA; 2 Center for Applied Energy Research, University of Kentucky, Lexington, KY 40511, USA

**Keywords:** hematite, potassium, Fisher–Tropsch, photocatalysis

## Abstract

Potassium is used extensively as a promoter with iron catalysts in Fisher–Tropsch synthesis, water–gas shift reactions, steam reforming, and alcohol synthesis. In this paper, the identification of potassium chemical states on the surface of iron catalysts is studied to improve our understanding of the catalytic system. Herein, potassium-doped iron oxide (α-Fe_2_O_3_) nanomaterials are synthesized under variable calcination temperatures (400–800 °C) using an incipient wetness impregnation method. The synthesis also varies the content of potassium nitrate deposited on superfine iron oxide with a diameter of 3 nm (Nanocat^®^) to reach atomic ratios of 100 Fe:*x* K (*x* = 0–5). The structure, composition, and properties of the synthesized materials are investigated by X-ray diffraction, differential scanning calorimetry, thermogravimetric analysis, Fourier-transform infrared, Raman spectroscopy, inductively coupled plasma-atomic emission spectroscopy, and X-ray photoelectron spectroscopy, as well as transmission electron microscopy, with energy-dispersive X-ray spectroscopy and selected area electron diffraction. The hematite phase of iron oxide retains its structure up to 700 °C without forming any new mixed phase. For compositions as high as 100 Fe:5 K, potassium nitrate remains stable up to 400 °C, but at 500 °C, it starts to decompose into nitrites and, at only 800 °C, it completely decomposes to potassium oxide (K_2_O) and a mixed phase, K_2_Fe_22_O_34_. The doping of potassium nitrate on the surface of α-Fe_2_O_3_ provides a new material with potential applications in Fisher–Tropsch catalysis, photocatalysis, and photoelectrochemical processes.

## 1. Introduction

Potassium has been used widely as a promoter for iron catalysts in Fisher–Tropsch synthesis, whereby syngas (CO + H_2_) is converted into liquid fuels [1,2,3,4]. As a promoter with iron catalysts, potassium increases CO chemisorption, but strongly inhibits hydrogen chemisorption in comparison to bare iron oxide [5,6]. Potassium donates electrons to adjacent iron centers that help to strengthen the Fe–C bonds while weakening the Fe–H bonds, increasing the adsorption of CO and decreasing the adsorption of H_2_, respectively [7,8]. This potassium-iron interaction and the superiority of potassium to other alkali or alkali earth metal promoters have been found to be advantageous in Fisher–Tropsch synthesis [9,10,11,12,13]. In addition, potassium is also used as a promoter with other catalysts (e.g., cobalt-based, aluminum-based, copper-based, manganese-based, etc.) for Fisher–Tropsch synthesis, hydrodesulfurization, steam reforming, methane (CH_4_) reforming, alcohol synthesis, alkene synthesis, water–gas-shift reactions, nitric oxide (NO_x_) removal, ammonia (NH_3_) synthesis, oxidation, etc. [10,12,14,15,16,17,18,19].

For the processes mentioned above, the incipient wetness impregnation method (IWIM) is often used to create catalysts by doping their surfaces with potassium. Potassium hydroxide (KOH) [16], or potassium salts of carbonate [11], bicarbonate [20], nitrate [21], and acetate [22], have been used to prepare potassium-doped catalysts by IWIM. Although a few different calcination temperatures at low potassium loadings have been explored [9,14,19], there has been no systematic study varying simultaneously both the potassium content and the temperature of calcination to generate the catalysts of interest. For example, the calcination of KOH with aluminum-magnesium catalysts at 400 °C has been used in the hydrodesulfurization of dibenzothiophene [16]. Similarly, for Fisher–Tropsch synthesis, potassium oxide (K_2_O) has been generated during the calcination (350–500 °C) of potassium carbonate (K_2_CO_3_) on iron [16], iron-manganese [9], iron-cobalt [19], and reduced graphene oxide-supported iron catalysts [18]. This K_2_CO_3_ precursor has also served to produce K_2_O on zinc-chromium-based catalysts, calcined at 400 °C, which are used for the synthesis of isobutanol [17]. Alternatively, the potassium hydrogen carbonate (KHCO_3_) precursor has been used on iron and iron-manganese catalysts, calcined at 300 °C, to create K_2_O for Fisher–Tropsch synthesis [20].

Interestingly, potassium nitrate (KNO_3_) has been calcined with gold-titanium-silicate [23] and cobalt-copper-titanate [24] at ≤350 °C with the objective of creating catalysts for converting carbon dioxide (CO_2_) into propanol and long-chain hydrocarbons, respectively. For applications in Fisher–Tropsch synthesis, KNO_3_ has been calcined in the range of 300–500 °C with iron-based catalysts [13,21,25]. Furthermore, in order to decompose NO_2_, the in situ generation of the K_2_O promoter was attempted over copper catalysts from KNO_3_, calcined at 400 °C [14]. However, the use of low loadings of KNO_3_ precursor to dope catalysts with small concentrations of K_2_O has been assumed to proceed at specific calcination temperatures, although typical instrumental limitations may have prevented the verification of the chemical and electronic states of interest.

An additional, almost unnoticed problem associated with calcination when it is used to create catalysts with low loadings of the potassium precursor is that the decomposition behavior of the precursor (e.g., KNO_3_) can change with increasing temperature. For example, at each temperature increment, the decomposition products that are generated can vary significantly, affecting the catalytic behavior of the material. Therefore, in this work, we perform a systematic study to optimize the calcination temperature of KNO_3_ (as the only precursor) on superfine iron oxide with a diameter of 3 nm (Nanocat^®^) and monitor the decomposition products. The surface-loaded potassium–iron oxides are synthesized by IWIM, with atomic ratios of 100 Fe:*x* K (*x* = 0, ½, 1, 2, 3, and 5). In other words, we study the chemical and electronic states generated from variable amounts of KNO_3_ precursor with a fixed iron content. The most highly potassium-doped sample, 100 Fe:5 K, was selected to characterize its calcination in air from 400 °C to 800 °C, to understand how the decomposition proceeds.

This work studies the effects of both potassium loading and calcination temperature on the structure and electronic state of synthesized potassium-doped iron oxide. Based on recent studies, the calcination temperature may affect the catalytic activity of iron oxide-based catalysts by changing the electronic and chemical state of potassium in the vicinity of iron oxide [26,27,28]. On the other hand, the changing composition of potassium may also affect active sites on the catalyst and, thereby, the catalytic performance [29,30,31]. An iron-based catalyst was synthesized using a potassium nitrate precursor with varying potassium loading and a calcination temperature of 400 or 500 °C to study the CO_2_ hydrogenation reaction [32,33,34]. The effect of potassium loading for the selective catalytic reduction of NO_x_ with an iron-based catalyst has been investigated [35]. Potassium promotion on iron catalysts obtained by pyrolysis has been explored for the purpose of ammonia synthesis [36]. However, and to the best of our knowledge, there is no systematic work available in the literature that has varied the calcination temperature of potassium-loaded iron oxide and simultaneously studied the electronic and structural properties of both components.

Overall, evaluating the influence of the promoter effects (e.g., metal salts [37]) in the environment of iron oxides can be a challenging experimental task, due to the large number of variables that need to be controlled during the synthesis (e.g., temperature, pressure, amount of promoter precursor, etc.). Simultaneously, there is a need to characterize low levels of the promoter and its structural and electronic properties [34,37,38,39]. Even small changes in the iron oxide phase, composition, and morphology (e.g., by the addition of structural (Al_2_O_3_) or electronic (K_2_O) promoters [34,38,39]) can affect the catalysts’ performance and the mechanism of catalysis for important processes such as the synthesis of ammonia and the Fisher–Tropsch process [38,39]. Therefore, to reach a better understanding of the catalytic performance and catalysis mechanism of potassium (promoter)-based iron oxide catalysts, it is important to know in detail the electronic state and structural parameters of both the promoter and catalyst reported in this work.

## 2. Materials and Methods

### 2.1. Preparation of Potassium-Doped Iron Oxides

Nanocat^®^ superfine iron oxide (Mach I Inc., King of Prussia, PA, USA, 99.3%) of hematite phase (α-Fe_2_O_3_) was dried at 110 °C for 3 h in an oven (Blue M, Single-Wall Transite Oven, White Deer, PA, USA). The raw material has a Brunauer–Emmett–Teller (BET) surface area of 244.24 m^2^ g^–1^, a particle size of 3 nm, and a bulk density of 0.05 g cm^–3^. For the potassium precursor, KNO_3_ was obtained commercially (Sigma-Aldrich, St. Louis, MO, USA, 99.0%). Both Nanocat^®^ and KNO_3_ were used to obtain a series of α-Fe_2_O_3_ materials loaded with potassium, following the IWIM. For the syntheses, 15.0 mL solutions of KNO_3_ were prepared in deionized water at concentrations of 0.0213, 0.0426, 0.0852, 0.1277, and 0.2130 M. Each solution was added slowly to 5.0 g of α-Fe_2_O_3_ placed in a rotating 250.0 mL round bottom flask using a customized West-type condenser provided with a polytetrafluoroethylene stopcock for dropwise addition. Rotation of the flask at a rate of 20 rpm was provided with a rotavapor (Brinkmann, Wood Dale, IL, USA, R110). The resulting materials were dried at 110 °C overnight in porcelain crucibles. These dried samples were then calcined in a muffle furnace (Thermo Scientific, Waltham, MA, USA, Lindberg) at 400 °C for 5 h in air. By varying the amount of KNO_3_ loaded, materials with atomic ratio 100 Fe:*x* K (*x* = 0, ½, 1, 2, 3, and 5) were obtained. In addition, the 100 Fe:5 K material was further calcined in air for 5 h at 500 °C, 600 °C, 700 °C, and 800 °C.

### 2.2. Characterization of Materials

Samples of 0.2500 g were weighed accurately into a Teflon MARSXpress vessel and 10.0 mL of nitric acid (HNO_3_, Acros Organics, Morris Plane, NJ, USA, ACS reagent, 65%) was added. The vessels were then closed and placed into a microwave digestion system (One-Touch Technology, Matthews, NC, USA, Mars 6), where the digestion was carried out with a power of 1.3 kW using a temperature ramp reaching 200 °C in 25 min, which was held at 200 °C for an extra 10 min. The digested samples were then allowed to cool for at least 3 h before opening the vessels. After cooling, the digested sample was augmented to a final volume of 10.0 mL with 10% nitric acid, from which 1.0 mL aliquots were diluted 10 times with 10% nitric acid to prepare stock solutions for potassium analysis. A further 1000-times dilution of the stock solution in 10% HNO_3_ was used for the iron analysis.

The analysis of potassium and iron in the previous samples was performed by inductively coupled plasma optical emission spectroscopy (ICP-OES, Varian, Santa Clara, CA, USA, Vista-PRO CCD Simultaneous). The analysis for iron considers an average for emission maxima at 234.35, 238.20, 239.56, and 259.94 nm, while for potassium, the maximum at 766.49 nm is used. Multi-elemental standards of iron and potassium (Ultra Scientific, Santa Clara, CA, USA, with each analyte at 1000 μg mL^–1^) were used to create calibration curves with solutions of concentration at ½, 1, 5, 10, and 50 ppm in 10% HNO_3_. Five parts per million (ppm) standards were used as check standards, and 1 ppm yttrium in 10% HNO_3_ served as an internal standard (CPI International, 1000 µg mL^–1^ in 2% HNO_3_). Samples were analyzed in triplicate. The operating conditions of the ICP-OES instrument were as follows: 1.20 kW radio-frequency power, 15.0 L min^–1^ plasma flow rate, 1.50 L min^–1^ auxiliary flow rate, 3 mL min^–1^ sample uptake, 35 s read delay, and 0.9 L min^–1^ nebulizer gas flow rate. 

The X-ray diffraction (XRD) patterns of the samples were obtained with B8 Advance Bruker AXS diffractometer with Cu Kα incident radiation (λ = 0.15418 nm). XRD data were recorded from 10° to 90°, at a scan rate of 1° min^–1^. Thin films of the materials were created by finely grinding the solids in an agate mortar, suspending the powders in water, depositing the suspensions on glass slides, and air-drying. The diffraction pattern of an empty sample holder was recorded and subtracted during the data processing.

A TA Instruments Q5000 was used for the thermogravimetric analysis (TGA) of samples heated under a nitrogen atmosphere, at a flow rate of 25 mL min^–1^ and a heating rate of 10 °C min^–1^ from 30 to 800 °C.

The X-ray photoelectron spectroscopy (XPS) surface analysis was recorded with a pass energy of 50 eV at a step size of 0.1 eV and X-ray beam size of 400 μm, using a Thermo Scientific spectrometer (K-Alpha) with an Al Kα anode (1486.6 eV photon energy, 300 W) excitation source.

The Raman spectra (200 scans) were recorded using a DXR Raman microscope (Thermo Scientific). A diode-pumped Nd:YVO_4_ laser was used as the excitation source (532 nm), with 2 mW power and a 50 μm aperture slit for Raman characterization.

Transmission electron microscopy (TEM) analysis was carried out using a Talos F200X instrument (Thermo Scientific) operated at an acceleration voltage of 200 kV. Images were recorded with Ceta 16M camera and Velox software (FEI). Selected-area electron diffraction patterns (SAED) and energy-dispersive X-ray spectroscopy (EDS) with four silicon drift detectors were also investigated from the electron micrographs. Before analysis, the as-prepared particles were dried in an oven for 24 h at 120 °C. The dry particles were then suspended in ethanol and sonicated for 30 min prior to being dropped onto a TEM grid with a lacy support film, which was allowed to air-dry for about 6 h.

## 3. Results and Discussion

### 3.1. Powder X-ray Diffraction Analysis

**Figure 1:**
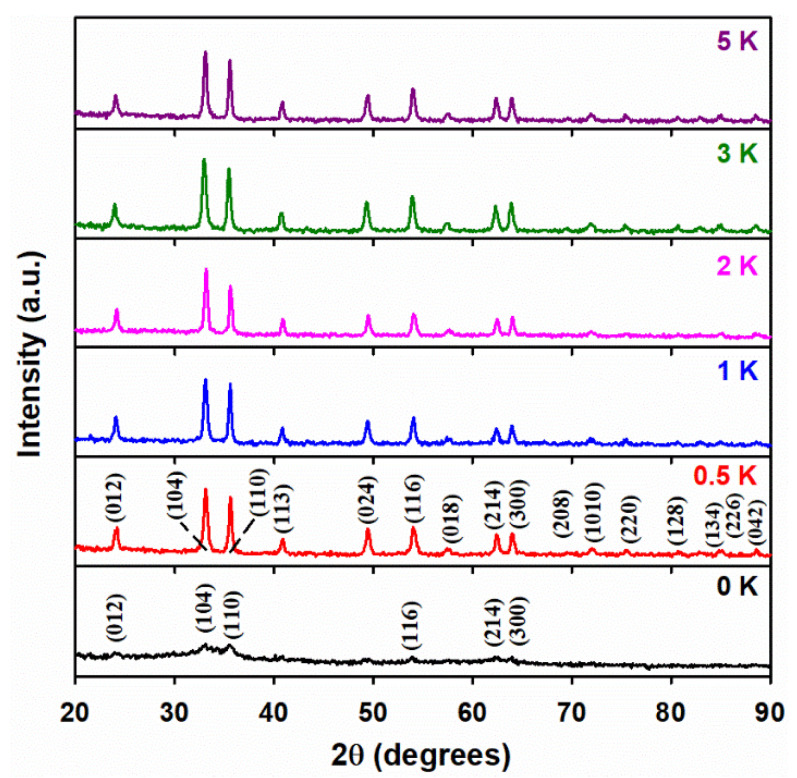
X-ray diffraction (XRD) patterns of 100 Fe:*x* K materials, with the atomic ratio of potassium (*x*) indicated in each panel. The assigned Miller indices match the hematite phase of iron oxide.

Figure 1 shows the XRD patterns of bare α-Fe_2_O_3_ and the materials doped with increasing potassium content under a calcination temperature of 400 °C. The diffraction peaks of bare α-Fe_2_O_3_ (100 Fe:0 K) centered at 24.2°, 33.1°, 35.6°, 54.1°, 62.5°, and 63.8° are labeled, respectively, as (012), (104), (110), (116), (214), and (300) on the reflections at the bottom of Figure 1, which reveal the crystalline phase of hematite. The low crystallinity of the undoped nanomaterial explains the low intensity and large peak broadening observed. The intensity of the previous reflections becomes larger for the K-doped materials with better-defined diffraction peaks in Figure 1, reflecting increased crystallinity. Moreover, all the peaks in the doped material are perfectly indexed as iron oxide with a hematite phase, with the rhombohedral lattice system (hexagonal axes, space group R3c), and with JCPDS card number #033-0664. The potassium-doped materials show the main characteristic peaks of α-Fe_2_O_3_ at 2θ values of 24.2°, 33.1°, 35.6°, 40.8°, 49.5°, 54.1°, 62.5°, 63.8°, 69.8°, 72.1°, 75.6°, 77.9°, 80.9°, 83.2°, 85.2°, and 88.7°, corresponding to diffraction from the planes (012), (104), (110), (113), (024), (116), (112), (214), (300), (208), (1010), (217), (036), (128), (0210), (134), and (226).

**Figure 2:**
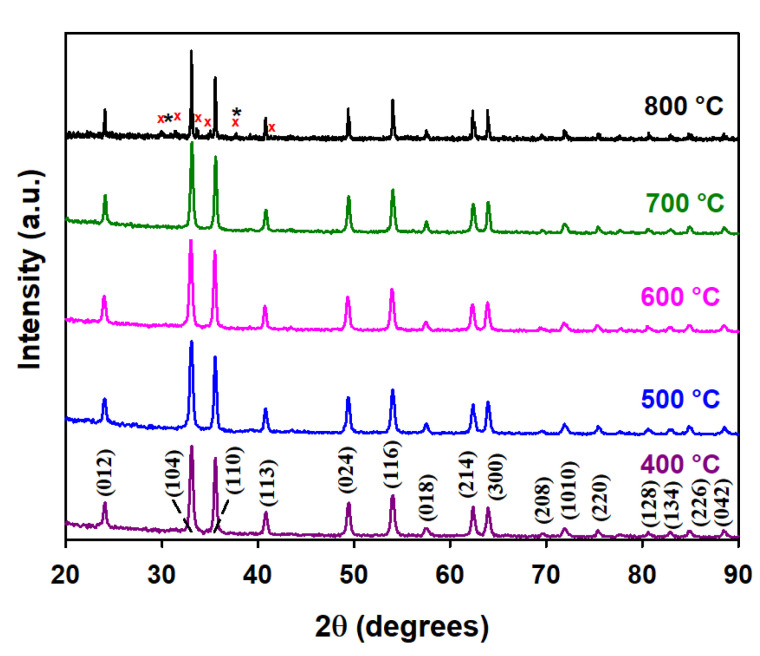
XRD patterns of the 100 Fe:5 K material calcined at the temperature indicated in each panel. The assigned Miller indices match the hematite phase of iron oxide. The peaks are marked as black asterisks (*) for K_2_O and as red crosses (×) for K_2_Fe_22_O_34_.

Figure 1 does not display any typical diffraction peaks of either KNO_3_ or KNO_2_, either because these species are well-dispersed over the surface and pores of α-Fe_2_O_3_ without forming detectable aggregates, or because very low concentrations of these species remain after IWIM. Similarly, the negative detection of K_2_O or any other mixed oxide phase(s) (Figure 1), even for the largest potassium loading (5 K), may be due to the stability of KNO_3_ at 400 °C. The previous hypotheses are verified below for the XRD patterns of 100 Fe:5 K material in Figure 2 (calcined at higher temperatures than in Figure 1), and by additional thermal spectroscopic analyses, which will be discussed later. No new diffraction peaks from hematite are observed upon increasing the calcination temperature from 400 to 700 °C (Figure 2), in agreement with the JCPDS card #033-0664. However, the calcination at 800 °C in Figure 2 shows peaks marked as (1) black asterisks for the cubic phase of K_2_O (JCPDS #027-0431) and (2) red crosses for the hexagonal phase of K_2_Fe_22_O_34,_ (JCPDS #031-1034), respectively. A close-up of the top panel of Figure 2 at 800 °C is displayed in Figure S1 (Supplementary Materials) with the corresponding position shown for the reflections of K_2_O and K_2_Fe_22_O_34_.

Potassium nitrate (KNO_3_) and its decomposition products, potassium nitrite (KNO_2_), and potassium superoxides, (KO_2_, JCPDS #010-0235) were not observed in Figure 2. K_2_O can also absorb water and form potassium hydroxide [40]. The in situ formation of potassium hydroxide (KOH) can also be ruled out by the comparison with JCPDS #015-0890. Therefore, the XRD pattern at 800 °C confirms the presence of hematite, potassium oxide, and potassium–iron mixed oxide. The relative compositions of these three species are calculated to be 82.68%, 1.83%, and 15.49%, respectively, using the semiquantitative peak area integration in Figure 2. The XRD patterns in Figure 2 become sharper with increasing calcination temperature, reflecting their increased crystallinity.

The effects of KNO_3_ loading and calcination temperature on the average crystallite size (*D*) of the K-doped α-Fe_2_O_3_ can be interpreted by applying Scherrer’s equation:

(1) $$D=\frac{k\;\lambda }{\beta \;cos\left(\theta \right)}\;,$$

to the major diffraction peak corresponding to the (104) plane, where *k* = 0.9 is the shape factor, *λ* = 0.15418 nm is the X-ray wavelength, *β* is the full width at half-maximum for the (104) reflection in radians, and *θ* is the Bragg angle. Table 1 displays how the crystallite sizes of the iron oxides vary with doping and calcination. Indeed, the crystallite size increases significantly with a small addition of potassium. For example, the 4.2 nm crystallite size of undoped α-Fe_2_O_3_ (100 Fe:0 K) increases to 16.1 nm for the 100 Fe:½ K material. For the further addition of potassium, the crystallite size remains almost the same at a constant calcination temperature of 400 °C. However, increasing calcination temperature leads to significant growth, up to a maximum of 36.6 nm at 800 °C.

The lattice parameters of the materials reported in Table 1 are calculated using equations that relate to the interplanar spacing, *d_hkl_* (Equation (2), for the Miller indices, *hkl*) [41], with Bragg’s law (Equation (3)):

(2) $$\frac{1}{d_{hkl}^{2}}=\frac{4}{3}\times \left(\frac{h^{2}+hk+l^{2}}{a^{2}}\right)+\frac{l^{2}}{c^{2}},$$

(3) $$2d_{hkl}\;sin\left(\theta \right)=n\;\lambda ,$$

where *n* is a positive integer and *λ* is the incident wavelength. Solving the equations for *d_110_* and *d_104_* for the planes (110) and (104) produces the lattice parameters of *a = b*, and *c*, which are reported in Table 1. Indeed, there is no significant change in the values of the lattice parameters *a* and *c* for undoped and K-doped α-Fe_2_O_3_ materials, calcined at different temperatures (Table 1).

Therefore, the crystallographic structure of hematite is largely preserved under the potassium loadings and calcination temperatures explored, indicating that potassium is not incorporated into the bulk defects of the α-Fe_2_O_3_ lattice. This is also evident from the larger cation size of K^+^ (138 pm) than Fe^3+^ (65 pm) [42]. Instead, the large K^+^ ions form K_2_O and a mixed phase of K-Fe oxides, K_2_Fe_22_O_34_.

### 3.2. Thermal Analysis

**Figure 3:**
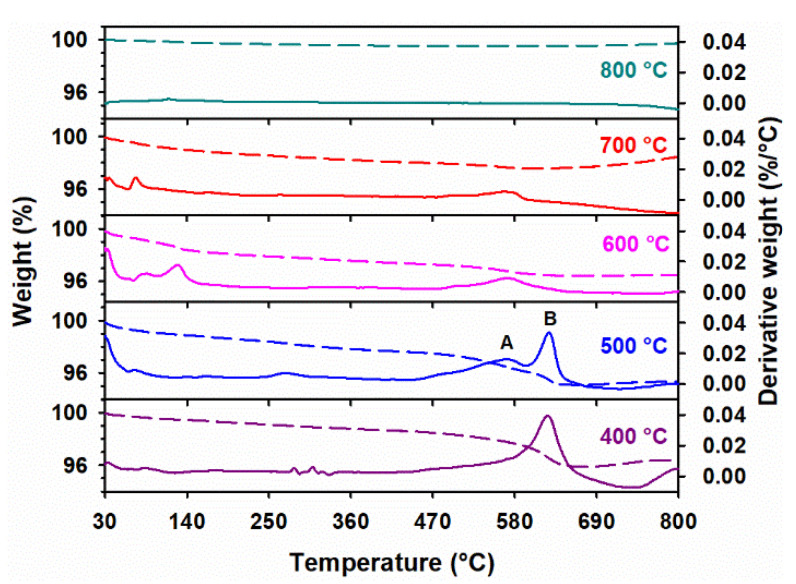
Thermogravimetric analysis (TGA, dashed line with the left vertical axis) and the first derivative of the thermogram (solid line with the right vertical axis) curves for 100 Fe:5 K material, at the calcination temperatures indicated in each panel. Peaks A and B correspond to the conversion of KNO_2_ to K_2_O, and of KNO_3_ to KNO_2_, respectively.

The TGA and first derivatives of the thermogram curves of the 100 Fe:5 K material at various calcination temperatures are displayed in Figure 3. For all the calcination temperatures investigated in Figure 3, there is a gradual loss of weight from the surface-adsorbed, interstitial, and bulk water of α-Fe_2_O_3_, with an appreciably strong thermal stability of up to 800 °C.

Two weight loss regions are identified in Figure 3 for the range of 450–600 °C (peak A) and 600–750 °C (peak B) in the first derivative of the thermogram curve of 100 Fe:5 K material. These two peaks are labeled for the panel at 500 °C in Figure 3, and they correspond to the decomposition of KNO_3_ to KNO_2_ (peak B at 624.5 °C) and KNO_2_ to K_2_O (peak A at 568.9 °C) [43,44,45]. Theoretically, while decomposing, KNO_2_ can also recombine with gaseous products (NO_2_ and NO) to reform KNO_3_ [45,46].

While pure KNO_3_ is known to be stable as a liquid (m.p. 334 °C) at least to 530 °C, the presence of a basic metal oxide, such as ZrO_2_, was shown to drastically reduce its decomposition temperature, e.g., to 340 °C [47], which is much lower than the temperature range of between 550 and 750 °C that has been used previously to characterize the equilibrium KNO_3_(*l*) ⇌ KNO_2_(*l*) + ½ O_2_(*g*) [48]. Thus, as the observed decomposition of potassium nitrate on the surface of iron oxide proceeds, peak B drops in size, generating potassium nitrite, which was previously absent in the system. Simultaneously with the previous event, peak A for the now formed potassium nitrite, decomposing into K_2_O and NO_x_ (NO + NO_2_), grows [45], to then disappear with all nitrogen species for the materials calcined between 600 and 800 °C.

The assignment of peaks A and B in Figure 3 is supported by similar observations over corundum, with a potassium-doped alumina phase [33]. Although peak B disappears for calcination at 600 °C and 700 °C, the presence of peak A at these temperatures (Figure 3) indicates that KNO_2_ is still present. The complete decomposition of KNO_3_ and KNO_2_ at 800 °C is noticeable in Figure 3. The corresponding thermal stability of the 100 Fe:*x* K materials in the range of 0 ≤ *x* ≤ 5 is reported in the Supplementary Materials (Figure S2), where all K-doped materials that are calcined at 400 °C show similar behavior, as described above.

### 3.3. Elemental Composition

The results from the ICP-OES analyses, reporting the elemental composition of the materials, are presented in Table 2 and show a good agreement between the experimental composition and the nominal values.

### 3.4. Raman Spectroscopy Analysis

**Figure 4:**
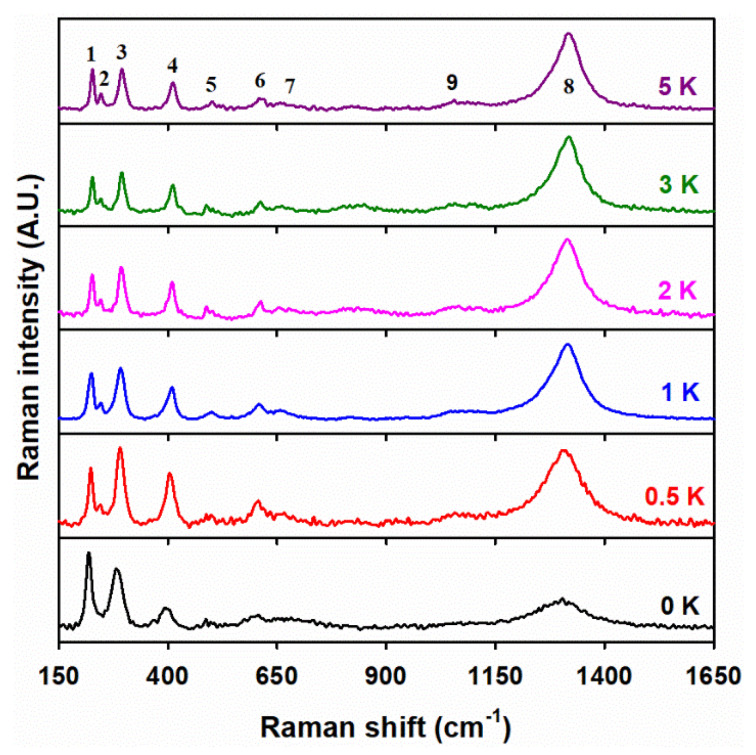
Raman spectra of 100 Fe:*x* K materials, with the atomic ratio of potassium (*x*) indicated in each panel. The numbers 1 to 8 correspond to modes of the hematite phase, while peak 9 relates to a mode of ${\mathrm{NO}}_{3}^{-}$ in the range of 980–1150 cm^–1^.

The Raman spectra of the undoped material in Figure 4 show the characteristic peaks (labeled 1 through 8) of the hematite phase in the 100 Fe:*x* K materials for increasing potassium content, calcined at 400 °C. Among these eight peaks for the undoped material, there are two *A*_1g_ modes centered at 219.3 cm^–1^ (peak 1) and 487.2 cm^–1^ (peak 5), four *E*_g_ modes at 244.6 cm^–1^ (peak 2), 281.3 cm^–1^ (peak 3), 400.0 cm^–1^ (peak 4), and 608.2 cm^–1^ (peak 6), one mode for the forbidden *E*_u_ mode, showing the presence of disorder in the hematite lattice at 661.8 cm^–1^ (peak 7), and one mode for the two-magnon scattering band centered at 1302.5 cm^–1^ (peak 8) [49,50,51,52]. For the K-doped materials, the peaks listed above are slightly shifted. For example, the hematite peaks for 100 Fe:5 K material in Figure 4 are centered at 221.7, 244.3, 286.2, 398.5, 488.0, 597.7, 661.5, and 1312.1 cm^–1^.

These shifts could be due to the phonon confinement effects from the nanoscale size of undoped hematite particles with a size of 3.0 nm (observed by TEM, as explained below). A changing chemical bonding effect by the potassium doping is excluded, as no shifts are registered for increasing potassium loadings. The previous observation agrees with the XRD data in Table 1 for the lack of potassium incorporation into the crystal lattice of α-Fe_2_O_3_. Figure 4 also confirms the presence of KNO_3_ in the doped materials, as evidenced by the Raman mode at 1055 cm^–1^ (peak 9) from the symmetric stretching vibration of ${\mathrm{NO}}_{3}^{-}$ [53]. Peak 9 becomes notable only in the 100 Fe:½ K material and increases its intensity for higher potassium loading. The Raman spectra for 100 Fe:5 K material calcined at higher temperatures, which are displayed in Figure S3 (Supplementary Materials), show that peak 9 starts to drop its intensity at 600 °C, and completely disappears at 700 °C. No change in the band position of the hematite peaks occurs for the increasing calcination temperature. No vibrational features for K_2_O or K_2_Fe_22_O_34_ could be observed by Raman spectroscopy due to the limitations of the technique, which are overcome by surface-sensitive XPS analysis.

### 3.5. XPS Analysis

**Figure 5:**
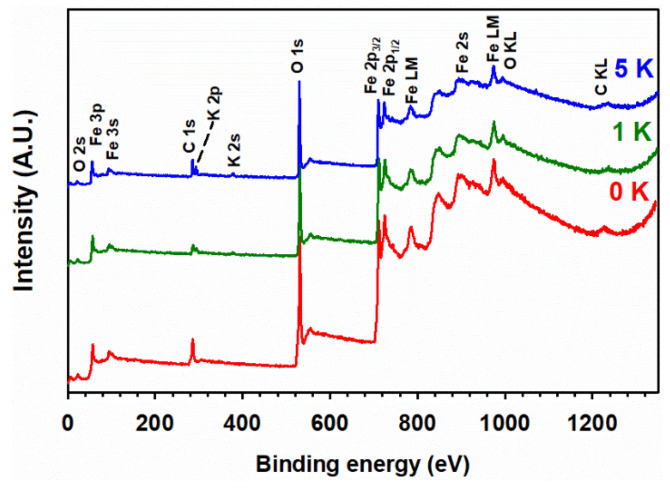
X-ray photoelectron (XPS) survey spectra for 100 Fe:*x* K materials with *x* = 0, 1, and 5, as indicated by the red, green, and blue traces, respectively. The peak assignment is based on the binding energy of photoelectron and Auger electron lines.

**Figure 6:**
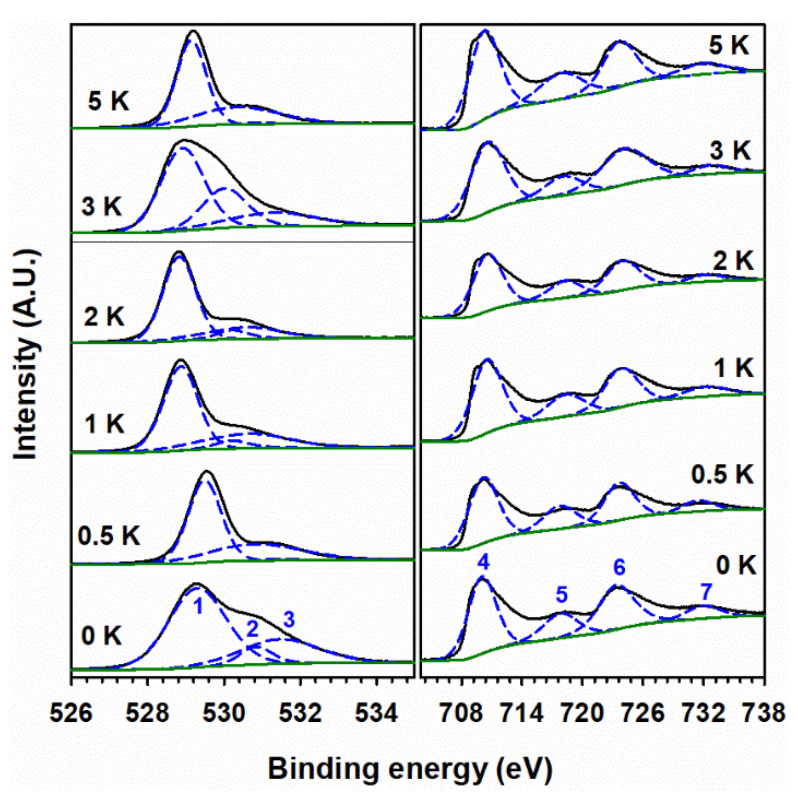
(Black trace) High-resolution XPS spectra of (**left panel**) O 1*s* and (**right panel**) Fe(III) 2*p* for 100 Fe:*x* K materials, calcined at 400 °C, with the atomic ratio of potassium (*x*) indicated in each panel. Dashed blue lines show the fitted peaks, numbered as follows: (1) O 1*s* for lattice oxides, (2) lattice hydroxyl, (3) surface hydroxyl, (4) Fe(III) 2*p*_3/2_, (5) Fe 2*p*_3/2_ satellite, (6) Fe(III) 2*p*_1/2_, and (7) Fe(III) 2*p*_1/2_ satellite. Solid green lines show the fitted background.

The surface composition of undoped and K-doped α-Fe_2_O_3_ after calcination at various temperatures is provided by XPS analysis. Qualitative elemental analysis is possible due to our analyzing the surveyed and high-resolution XPS spectra, which yield the electronic state and chemical bonding of the elements. For example, the XPS survey spectra of 100 Fe:*x* K for *x* = 0, 1, and 5 are shown in Figure 5. While undoped α-Fe_2_O_3_ only shows oxygen and iron (disregarding the presence of signals from adventitious carbon), the doped materials (e.g., for 1 K and 5 K in Figure 5) additionally display the presence of potassium; however, the nitrogen signals are too small and are only detected in the high-resolution XPS spectra. The left panel of Figure 6 shows the high-resolution XPS spectra for O 1*s*, with peaks centered at 529.3, 530.9, and 531.5 eV in the 100 Fe:0 K material, corresponding to the lattice oxygen binding with Fe(III) (denoted as Fe–O and labeled as peak 1) in α-Fe_2_O_3_, lattice hydroxyl (peak 2), and surface hydroxyl (peak 3), respectively.

With K-doping, the O 1*s* XPS spectra do not show any new features of oxygen related to a possible K–O bond. The high-resolution XPS spectra of Fe 2*p* (right panel in Figure 6) show two distinct, intense peaks at the binding energies of 710.1 eV and 723.5 eV, which correspond to Fe 2*p*_3/2_ (peak 4) and Fe 2*p*_1/2_ (peak 6), respectively. The doublet (peaks 4 and 6) arises due to the spin-orbit coupling in the Fe 2*p* states. There are also two additional satellite peaks at binding energies of 718.0 eV and 732.1 eV, assigned to Fe 2*p*_3/2_ (peak 5) and Fe 2*p*_1/2_ (peak 7), respectively. These features of Fe 2*p* are in good agreement with the Fe(III) states reported for α-Fe_2_O_3_ (hematite) [41,54,55]. The presence of Fe(II) 2*p* states was discarded, as there are neither spin-orbit splitting nor satellite peaks for Fe(II) states in both doped and undoped materials. The binding energies of peaks 1–7 are not shifted in the K-doped materials, discarding the incorporation of potassium into the lattice of α-Fe_2_O_3_.

**Figure 7:**
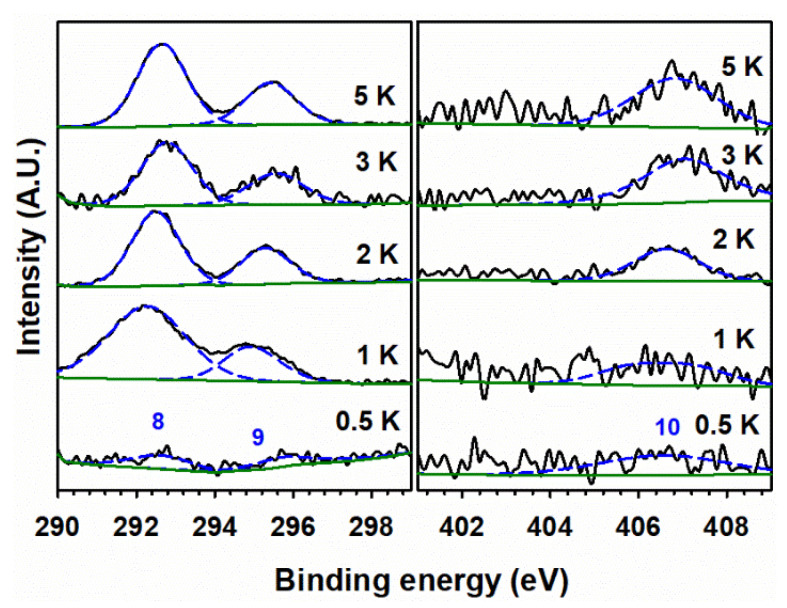
(Black trace) High-resolution XPS spectra of (**left panel**) K 2*p* and (**right panel**) N 1*s* for 100 Fe:*x* K materials, calcined at 400 °C, with the atomic ratio of potassium (*x*) indicated in each panel. (Dashed blue) Fitted peaks numbered (8) K 2*p*_3/2_, (9) K 2*p*_1/2_, and (10) nitrate. (Solid green) Fitted background.

The high-resolution XPS spectra for potassium in the left panel of Figure 7 show two peaks at the binding energies of 292.7 and 295.4 eV for K 2*p*_3/2_ (peak 8) and K 2*p*_1/2_ (peak 9), respectively. The binding energy difference of 2.7 eV for peaks 8 and 9 is due to the spin-orbit coupling of potassium cations in potassium nitrates [56]. No systematic shift in the binding energy of K 2*p* has been observed in these spectra, which indicates that the added potassium is not interacting chemically with α-Fe_2_O_3_. The presence of nitrate, remaining at this low calcination temperature, is also confirmed in the high-resolution XPS spectra for nitrogen (right panel of Figure 7) by peak 10 at 406.8 eV [57]. Peak 10 is hard to perceive in the 100 Fe:½ K material because of the very low concentration of nitrogen that is nominally present as KNO_3_.

**Figure 8:**
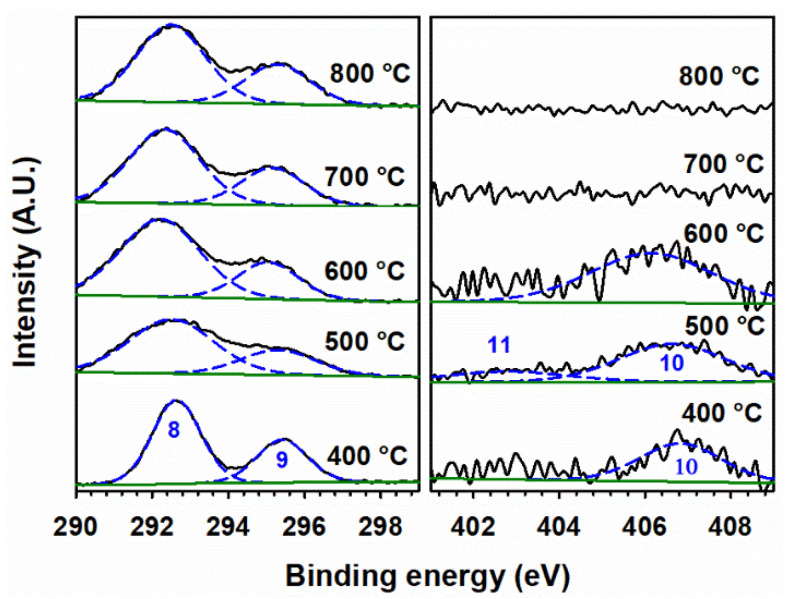
(Black trace) High-resolution XPS spectra of (**left panel**) K 2*p* and (**right panel**) N 1*s* for 100 Fe:5 K material, calcined at the indicated temperatures. (Dashed blue) Fitted peaks numbered (8) K 2*p*_3/2_, (9) K 2*p*_1/2_, (10) nitrate, and (11) nitrite. (Solid green) Fitted background.

**Figure 9:**
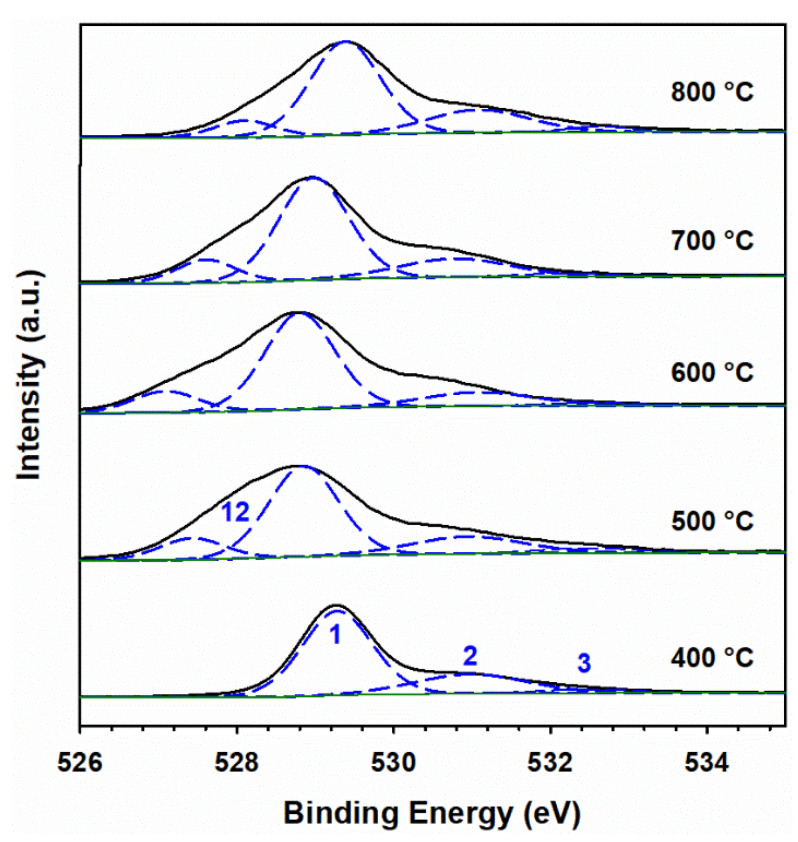
(Black trace) High-resolution XPS spectra of O 1*s* for 100 Fe:5 K material, calcined at the temperature specified in each panel. (Dashed blue) Fitted peaks numbered (1) O 1*s* for lattice iron oxides, (2) lattice hydroxyl, (3) surface hydroxyl, and (12) K–O bonded species. (Solid green) Fitted background.

The observation of peak 10 for nitrate in these doped materials indicates that KNO_3_ did not decompose when calcined at 400 °C. Figure 8 presents high-resolution XPS spectra for 100 Fe:5 K with K 2*p* (peaks 8 and 9) and N *1s* (peak 10, for nitrate) signals at various calcination temperatures. Peak 10 is observed at 400, 500, and 600 °C. In addition, a peak at 402.8 eV for the nitrite (peak 11) [57] is clearly observed at 500 °C, confirming its production as a result of the decomposition of nitrate. The binding energies of peaks for spin-orbit coupling and their corresponding satellite peaks of Fe 2*p* neither show a shift with calcination temperature nor demonstrate the presence of new peaks in the high-resolution XPS spectra (Figure S4, Supplementary Materials). Thus, the starting hematite phase is maintained by α-Fe_2_O_3_ for all calcination temperatures and compositions, but a solid solution is formed at 800 °C, as evidenced by the peaks marked with a red cross in Figure 2. Even at 800 °C (Figure S4, Supplementary Materials), no change in the oxidation state of iron is registered by XPS. However, a small contribution of iron(II) 2*p* [58] may be observed in the high-resolution XPS spectra for the samples calcined at 500 °C and 600 °C (Figure S4, Supplementary Materials). Calcination at higher temperatures can create oxygen vacancies in the iron oxide lattice, which leaves two electrons per oxygen atom [59]. Therefore, the introduction of two electrons can reduce iron(III) to iron(II). The absence of any nitrogen-bonded species in the right panel of Figure 8 proves that the complete decomposition of KNO_3_ and KNO_2_ occurs at 700 °C and 800 °C, which produces K_2_O and K_2_Fe_22_O_34_ (as seen in Figure 2). The hypothetical production of metallic potassium at high calcination temperature is discarded as no K 2*p* doublet, for K 2*p*_3/2_ and K 2*p*_1/2_, is registered at 295 and 298 eV, respectively [56]. The O 1*s* fitting for the sample calcined at various temperatures is shown in Figure 9. The peak fitting was carried out by keeping the full-width half-maximum of the lattice iron oxygen constant in all calcined materials [60]. An additional peak at lower binding energy could be assigned to the potassium-bonded oxygen species (K–O), likely provided by NO_3_^–^, in the doped samples. Table S1 (Supplementary Materials) provides the surface ratio of potassium to iron content in experiments under variable potassium loading and calcination temperatures, which value is obtained by the integration of the areas under the fitted peaks for K 2*p*_3/2_ and Fe 2*p*_3/2_ in XPS in Figure 6, Figure 7 and Figure 8.

### 3.6. TEM and EDS Analyses

**Figure 10:**
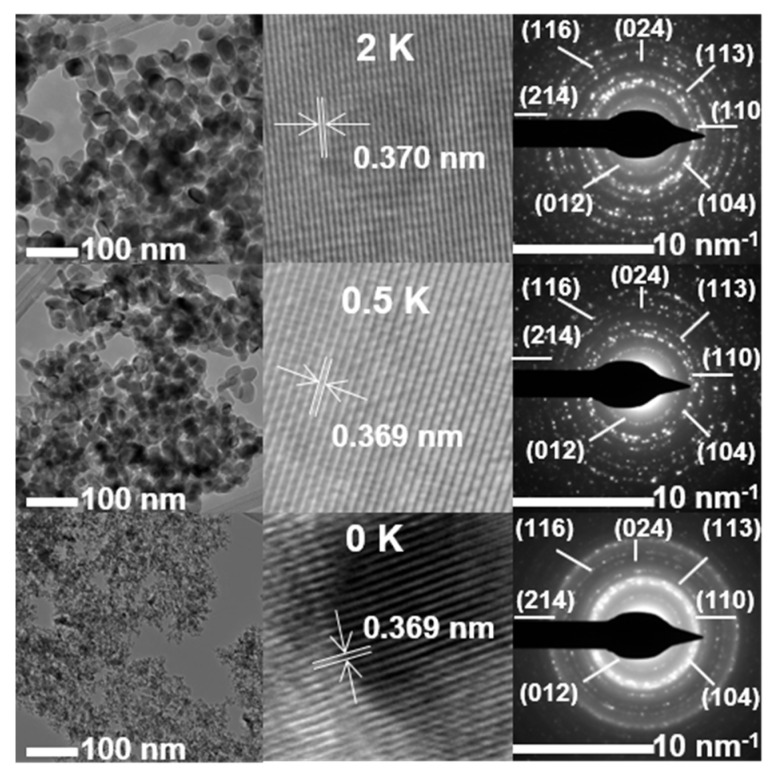
(From left to right) Transmission electron microscopy (TEM), high-resolution TEM (HRTEM), and selected area electron diffraction (SAED) patterns of (from top to bottom) 100 Fe:2 K, 100 Fe:½ K, and 100 Fe:0 K materials, calcined at 400 °C.

The size, morphology, and microstructure of the materials are characterized using TEM and high-resolution TEM (HR-TEM). Individual hexagonal particles of undoped and K-doped α-Fe_2_O_3_ are shown in Figure 10. The particle sizes of the materials in Figure 10 are reported in Table 3, based on the distribution analysis of individual particles in the histograms in Figure S5 (Supplementary Materials). For undoped α-Fe_2_O_3_, the average diameter of the particles is about 2.56 nm.

The undoped particles in Figure 10, with an average particle diameter of 2.51 nm, grow to 15.2 and 17.4 nm after the addition of ½ K and 2 K, respectively (Table 3), without any further significant change for 5 K when the calcination was 400 °C. The increase in particle size indicates that the addition of potassium results in larger crystals. This observed behavior by TEM (Table 3) follows the same trend for the crystallite size obtained by XRD (Table 1). The HRTEM images show good crystallization, with well-defined lattice fringes at an interplanar spacing of 0.369 nm, which is consistent with a *d*_012_ of α-Fe_2_O_3_ within ±0.002 nm for the materials with or without potassium (Figure S6, Supplementary Materials). These *d*_012_ values correspond to the reciprocal of the radius of each diffraction ring in the SAED patterns and are in excellent agreement with the X-ray crystallographic data (JCPDS #033-0664 with *d*_012_ = 0.368 nm). In addition, the interplanar spacings of other major diffraction peaks remain unchanged for both undoped and doped materials (Figure S6, Supplementary Materials). Furthermore, the SAED patterns (Figure 10) only show the continuous ring patterns for the α-Fe_2_O_3_ phase, without any additional diffraction spots and rings, even after potassium doping.

Table 3 also reports the potassium contents on the surface of the materials, which are obtained during TEM by EDS analysis, followed by the integration of the peak areas. For low-potassium doping, the experimental composition of the surface is close to the nominal value (Table 3), but for increasing nominal potassium content, there is a significant deviation due to heterogeneous doping.

**Figure 11:**
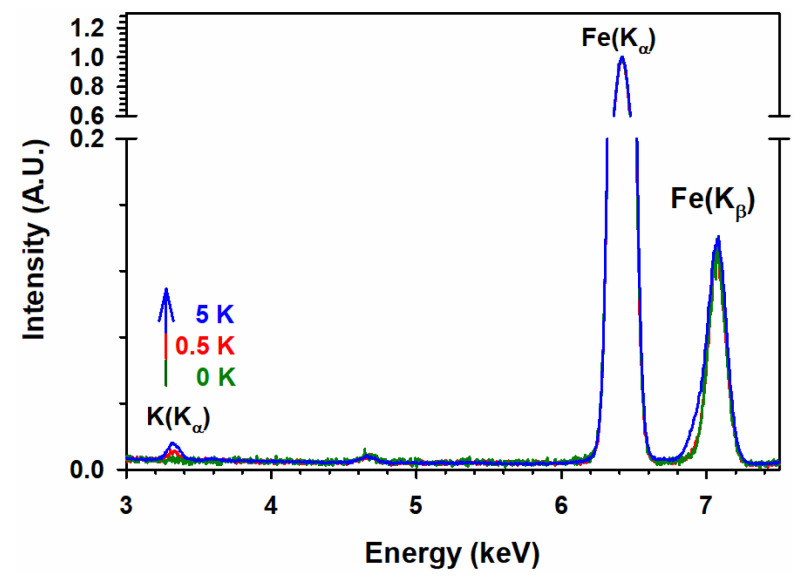
Energy-dispersive X-ray spectroscopy (EDS) spectra of 100 Fe:*x* K materials for potassium contents (*x*) of (green) 0 K, (red) ½ K, and (blue) 5 K for the K (Kα) emission line.

**Figure 12:**
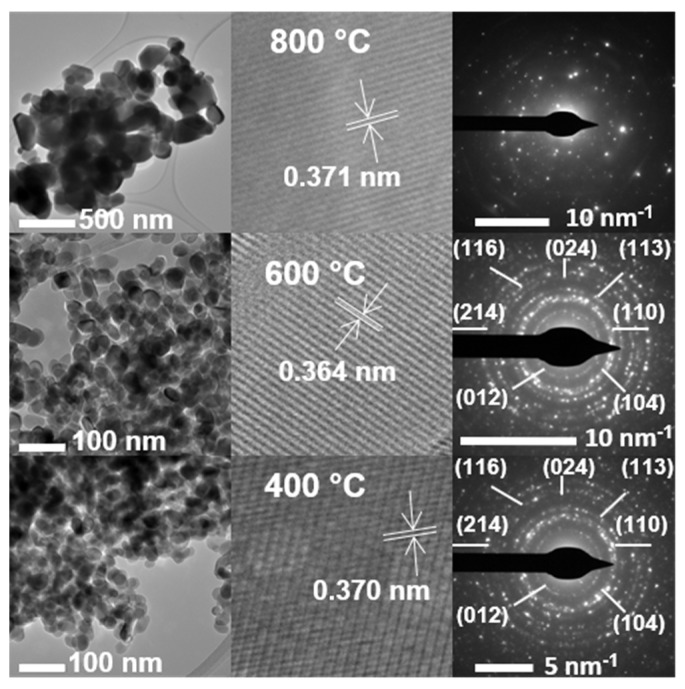
(From left to right) TEM, HRTEM, and SAED patterns of 100 Fe:5 K calcined at (from top to bottom), 800, 600, and 400 °C.

The compositional analysis of iron and potassium using ICP-OES matches the nominal content. However, the EDS analysis shows a significant deviation from the nominal values. As described earlier in XRD and Raman analysis, potassium ion is not incorporated into the iron oxide lattice due to the larger size of K^+^ than Fe^3+^. Therefore, potassium ions reside on the surface of iron oxide. Thus, such a deviation of potassium content in EDS analysis relative to the nominal composition may simply reflect a limitation of this technique. Moreover, during EDS analysis, potassium content was calculated from selected heterogeneous particles that, on average, have a lower potassium content than iron content. Hence, EDS provides more qualitative information about the potassium content [61,62] than IPC-OES. On the other hand, ICP-OES analyzes the bulk potassium content. Therefore, the results from ICP-OES represent the most reliable quantitative information [63]. Figure 11 shows examples for the increasing potassium content on the surface of 100 Fe:*x* K for *x* = 0, ½, and 5 K, as registered by EDS for the K (Kα) line. TEM images for 100 Fe:5 K material calcined at various temperatures are displayed in Figure 12, and the corresponding analysis of their size distribution is provided in Table 3 and Figure S5 (Supplementary Materials). The larger particle size, with increasing calcination temperature, is simply due to the agglomeration of particles. At the microscopic level, the high temperature enlarges the grain boundaries and improves mass transport, consequently allowing the particles to grow.

At the macroscopic level, the increase in particle size can be ascribed to the reduction in total surface energy caused by the calcination temperature. The calcined 100 Fe:5 K material retains the hematite phase up to 700 °C (see the XRD and Raman spectra above) without forming any new phase, as shown in the SAED patterns, for example at 600 °C in Figure 12, with unchanged interplanar spacings. The SAED patterns also reflect the polycrystallinity of the materials. For calcination at 800 °C, it was impossible to observe any clear rings in the SAED spectrum of 100 Fe:5 K, due to the large, agglomerated crystals. However, the few SAED spots that could still be assigned at 800 °C are accurately indexed by the hematite phase of α-Fe_2_O_3_.

## 4. Conclusions

The hematite phase of the materials remains stable upon the addition of potassium nitrate for iron oxide, with atomic compositions as high as 100 Fe:5 K up to 700 °C. However, XRD confirms that at 800 °C, two new phases are formed, K_2_O and K_2_Fe_22_O_34_. The transformation of KNO_3_ into KNO_2_ starts at 500 °C, and these species completely decompose at 800 °C. The current study verifies the finding that the remaining potassium produces K_2_O at 700 and 800 °C. This work confirms the importance of studying the chemical and electronic states of alkali metals such as potassium dopants in the host materials before using them as catalysts. We recommend the 100 Fe:5 K material calcined at 800 °C for catalytic applications of K_2_O/α-Fe_2_O_3_. Future work with potassium nitrate-doped iron oxide in the catalysis and photocatalysis fields can potentially improve multiple processes, if explored carefully, by ensuring the state of potassium species.

## Figures and Tables

**Table 1:** Crystallite size (*D*) and lattice parameters (*a* and *b*) for variable potassium loading (*x*) and calcination temperature (*T*).

X ^i^	T (°C)	D (nm) ^ii^	a (Å) ^iii^	c (Å) ^iii^
0	400	4.15	5.059	13.746
½	400	16.1	5.042	13.782
1	400	18.1	5.047	13.787
2	400	19.3	5.040	13.761
3	400	17.1	5.063	13.844
5	400	20.0	5.044	13.794
5	500	20.0	5.051	13.807
5	600	21.2	5.058	13.829
5	700	23.6	5.046	13.780
5	800	36.6	5.067	13.841

^i^ Atomic ratio of potassium for 100 Fe:*x* K (0 ≤ *x* ≤ 5), ^ii^ from Scherrer’s equation, ^iii^ from the interplanar spacing and Bragg’s law.

**Table 2:** Bulk compositional analysis of the materials.

Nominal ^iv^	Experimental ^v^
100 Fe:0 K	100 Fe:0 K
100 Fe:½ K	100 Fe:0.548(2) K
100 Fe:1 K	100 Fe:1.275(3) K
100 Fe:2 K	100 Fe:2.750(5) K
100 Fe:3 K	100 Fe:3.209(13) K
100 Fe:5 K	100 Fe:6.413(56) K

^iv^ Atomic ratio for fixed iron content, ^v^ from ICP-OES measurements.

**Table 3:** The average diameter of α-Fe_2_O_3_ particles and surface potassium content (*x*) for selected materials and calcination temperatures (*T*).

Material	*T* (°C)	Diameter (nm) ^vi^	*X* ^vi^
100 Fe:0 K	400	2.56	0.00
100 Fe:½ K	400	15.4	0.48
100 Fe:2 K	400	17.5	0.85
100 Fe:5 K	400	17.6	0.93
100 Fe:5 K	600	20.0	2.80
100 Fe:5 K	800	135.3	1.43

^vi^ Diameter calculated from the Gaussian fittings in the histogram obtained from TEM images. ^vii^ Potassium content from the integration of K(Kα) line in the EDS spectra.

## Data Availability

The data presented in this study are available upon reasonable request from the corresponding author.

## Supplementary Materials

The following supporting information can be downloaded at: https://www.mdpi.com/article/10.3390/ma15207378/s1, Additional experimental methods, additional results, and discussion; and Figures S1–S10 and Table S1 are in the Supplementary Materials files. References [64,65] are cited in the Supplementary Materials files.

## Abbreviations

DSC, Differential Scanning Calorimetry; ICP-OES, Inductively Coupled Plasma-Optical Emission Spectroscopy; IWIM, Incipient Wetness Impregnation Method; SAED, Selected Area Electron Diffraction; TEM, Transmission Electron Microscopy; TGA Thermogravimetric Analysis; XRD, X-ray Diffraction; XPS, X-ray Photoelectron Spectroscopy.
