# Supplementary material for: Chemical State of Potassium on the Surface of Iron Oxides: Effects of Potassium Precursor Concentration and Calcination Temperature
Source: Materials (Basel). 2022 Oct 21;15(20):7378. doi: 10.3390/ma15207378 (PMC9610504; doi:10.3390/ma15207378)
Supplement: Supplementary file 1 [file materials-15-07378-s001.zip › materials-1941824-supplementary.pdf]

materials

MDPI

Supplementary for Article

Chemical State of Potassium on the Surface of Iron Oxides: Effects of Potassium Precursor Concentration and Calcination Temperature

Md. Ariful Hoque [1], Marcelo I. Guzman [1,*], John P. Selegue [1] and Muthu Kumaran Gnanamani [2]

[1] Department of Chemistry, University of Kentucky, Lexington, KY 40506, USA
[2] Center for Applied Energy Research, University of Kentucky, Lexington, KY 40511, USA
* Correspondence: marcelo.guzman@uky.edu; Tel.: +1-(859)-3232892

Abstract: Potassium is used extensively as a promoter with iron catalysts in Fisher-Tropsch synthesis, water-gas shift reactions, steam reforming, and alcohol synthesis. The identification of potassium chemical states on the surface of iron catalysts is studied to improve the understanding of the catalytic system. Herein, potassium-doped iron oxides (α-Fe_2O_3) nanomaterials are synthesized under variable calcination temperatures (400–800 °C) using an incipient-wetness impregnation method. The synthesis also varies the content of potassium nitrate deposited on superfine iron oxide with diameter of 3 nm (Nanocat) to reach atomic ratios of 100 Fe:x K (x = 0–5). The structure, composition, and properties of the synthesized materials are investigated by X-ray diffraction, differential scanning calorimetry, thermogravimetric analysis, Fourier-transform infrared, Raman, - inductively coupled plasma-atomic emission and X-ray photoelectron spectroscopies, as well as transmission electron microscopy with energy dispersive X-ray spectroscopy and selected area electron diffraction. The hematite phase of iron oxide retains its structure up to 700 °C without forming any new mixed phase. For composition as high as 100 Fe:5 K, potassium nitrate remains stable up to 400 °C, but at 500 °C starts to decompose into nitrites, and only at 800 °C completely decomposes to potassium oxide (K_2O) and a mixed phase, $K_2Fe_{22}O_{34}$. The doping of potassium nitrate on the surface of α-Fe_2O_3 provides a new material with potential applications in Fisher-Tropsch catalysis, photocatalysis and photoelectrochemical processes.

Keywords: hematite; potassium, Fisher-Tropsch; photocatalysis

Citation: Hoque, M.A.; Guzman, M.I.; Selegue, J.P.; Gnanamani, M.K. Chemical State of Potassium on the Surface of Iron Oxides: Effects of Potassium Precursor Concentration and Calcination Temperature. *Materials* **2022**, *15*, 7378. https://doi.org/10.3390/ma15207378

Academic Editor: Stefano Agnoli

Received: 14 September 2022
Accepted: 19 October 2022
Published: 24 October 2022

Publisher's Note: MDPI stays neutral with regard to jurisdictional claims in published maps and institutional affiliations.

Copyright: © 2022 by the author. Licensee MDPI, Basel, Switzerland. This article is an open access article distributed under the terms and conditions of the Creative Commons Attribution (CC BY) license (https://creativecommons.org/licenses/by/4.0/).

Additional Experimental Methods. The differential scanning calorimetric (DSC) analyses of the samples were performed using a differential calorimetric analyzer (Q20, TA instruments). The samples were heated under nitrogen atmosphere under a flow rate of 50 mL/min with a heating rate of 10 °C/minute from 30 °C to 350 °C.

Fourier-transform infrared spectroscopic (FT-IR) analysis was performed with a FT-IR spectrophotometer (Nicolet 6700 FT-IR, Thermo Scientific) using a smart iTR™ attenuated total reflectance (ATR) sampling accessory with diamond / ZnSe crystal. The spectra were recorded in a range of 500 – 4000 cm^{-1} with a spectral resolution of 1 cm^{-1} and averaged over 256 scans.

Additional Results and Discussion. DSC curves also confirm the presence of undecomposed potassium nitrate in the samples. The orthorhombic to rhombohedral phase transition of potassium nitrate occurs at 135 °C and it starts to melt at approximately 315 °C [1,2]. On the other hand, iron oxide (hematite) is stable to 1300 °C [3]. Two weak endothermic peaks, (peak A) the phase transition of potassium nitrate centered at 133.9 °C and (peak B) melting of potassium nitrate centered at 313.3 °C are found in the DSC curve of 100 Fe:3 K samples as shown in Figure S7. However, with all other low potassium-doped samples such peaks of potassium nitrate are not observed, which may be due to the very low concentration of potassium nitrate. The intensity of such peaks increases for 100 Fe:5

K samples. To investigate the decomposition features of potassium nitrate, the DSC curves of 100 Fe:5 K samples with calcination are depicted in Figure S8. The absence of peak A (133.9 °C) in the DSC curve of samples calcined at 500 °C confirms that the phase transition of potassium nitrate from its most stable orthorhombic to rhombohedral happens at calcination temperature of 500 °C. However, the potassium nitrate is still present in the samples since the peak B (313.3 °C) is present in the DSC curves. The absence of 313.3 °C peak in the samples calcined at higher temperature indicates that either all the potassium nitrate decomposed or there may be a small amount of rhombohedral phase below the detection limit of DSC.

Potassium nitrate shows characteristics of N–O stretching vibration centered at 1351 cm^{-1} [4]. At low doping concentration of potassium nitrate, the band of N–O stretching vibration (peak C) for potassium nitrate was not found in the IR spectrum in Figure S9, probably due to very small potassium nitrate content. However, this band starts to appear in the spectrum for 100 Fe:3 K and intensity increases for 100 Fe:5 K. IR spectra of 100 Fe:5 K sample with various calcination temperatures are shown in Figure S10. The potassium nitrate N–O stretching vibration starts to disappear at 600 °C and completely disappears at 800 °C. The possible decomposition products of potassium nitrate are potassium nitrite and potassium oxides, but N–O stretching (potassium nitrite) and K–O stretching (potassium oxide) bands are not observed in the spectra. IR bands related to iron oxides are not seen due to the limitation of the instruments.

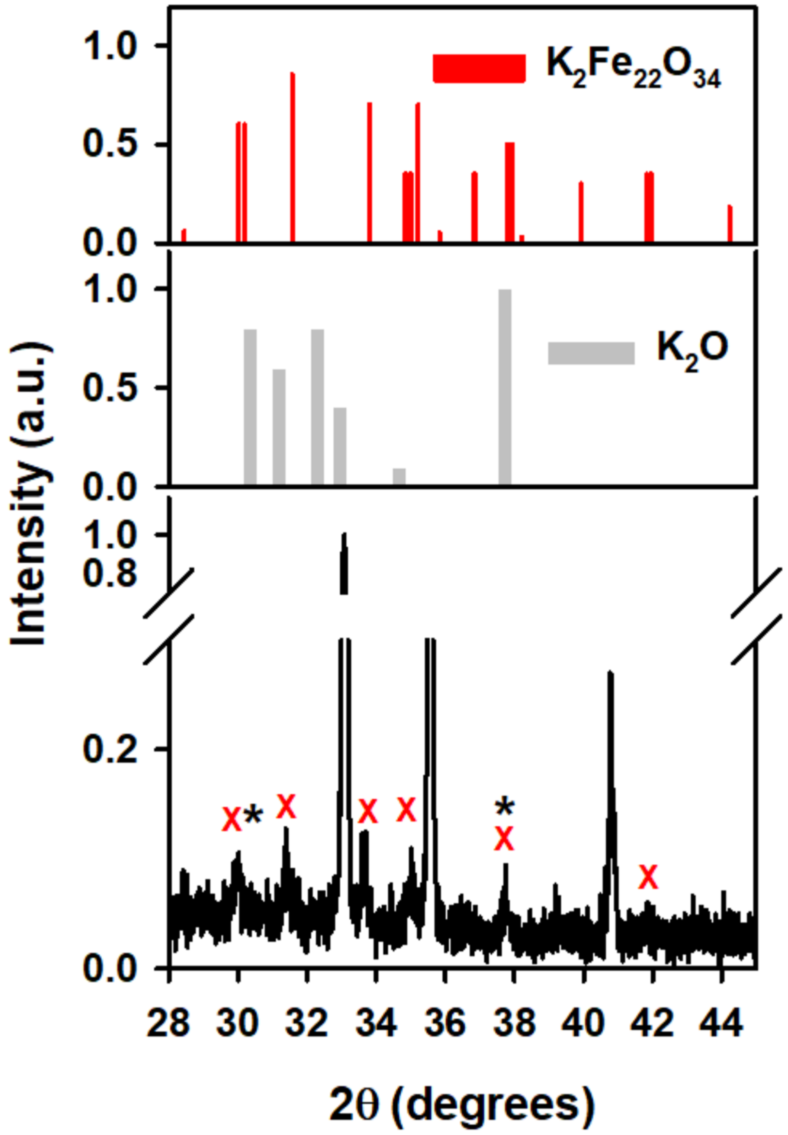

Figure S1. (Bottom) XRD pattern of 100 Fe:5 K material calcined at 800 °C. Assigned Miller indices match the hematite phase of iron oxide. Peaks are marked as black asterisks (*) for JCPDS # 027-0431 matches of K_2O in the center panel, and red crosses ( ) for JCPDS # 031-1034 matches of $K_2Fe_{22}O_{34}$ in the top panel.

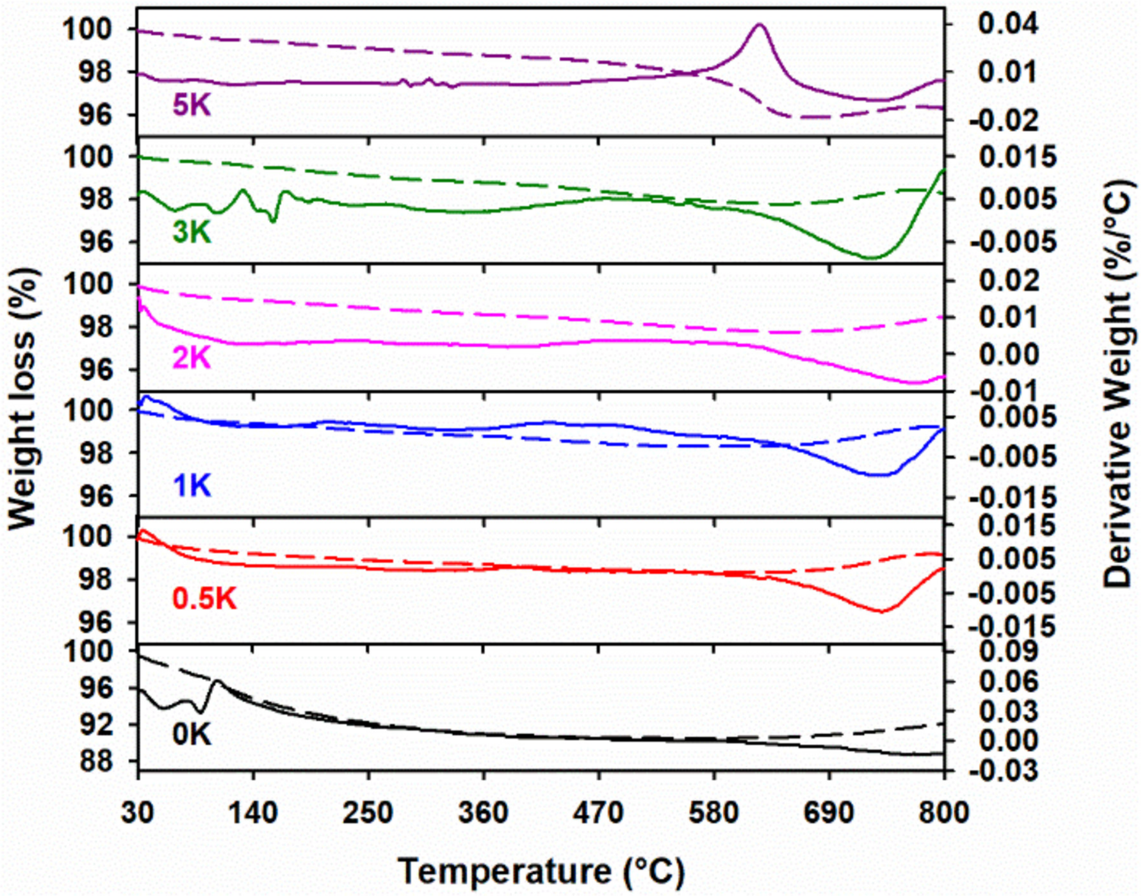

Figure S2. Thermogravimetric analysis (TGA, dashed line with the left vertical axis) and first derivative of thermogram (solid line with the right vertical axis) curves for 100 Fe:x K materials (calcined at 400 °C) with potassium loading (x) indicated in each panel. .

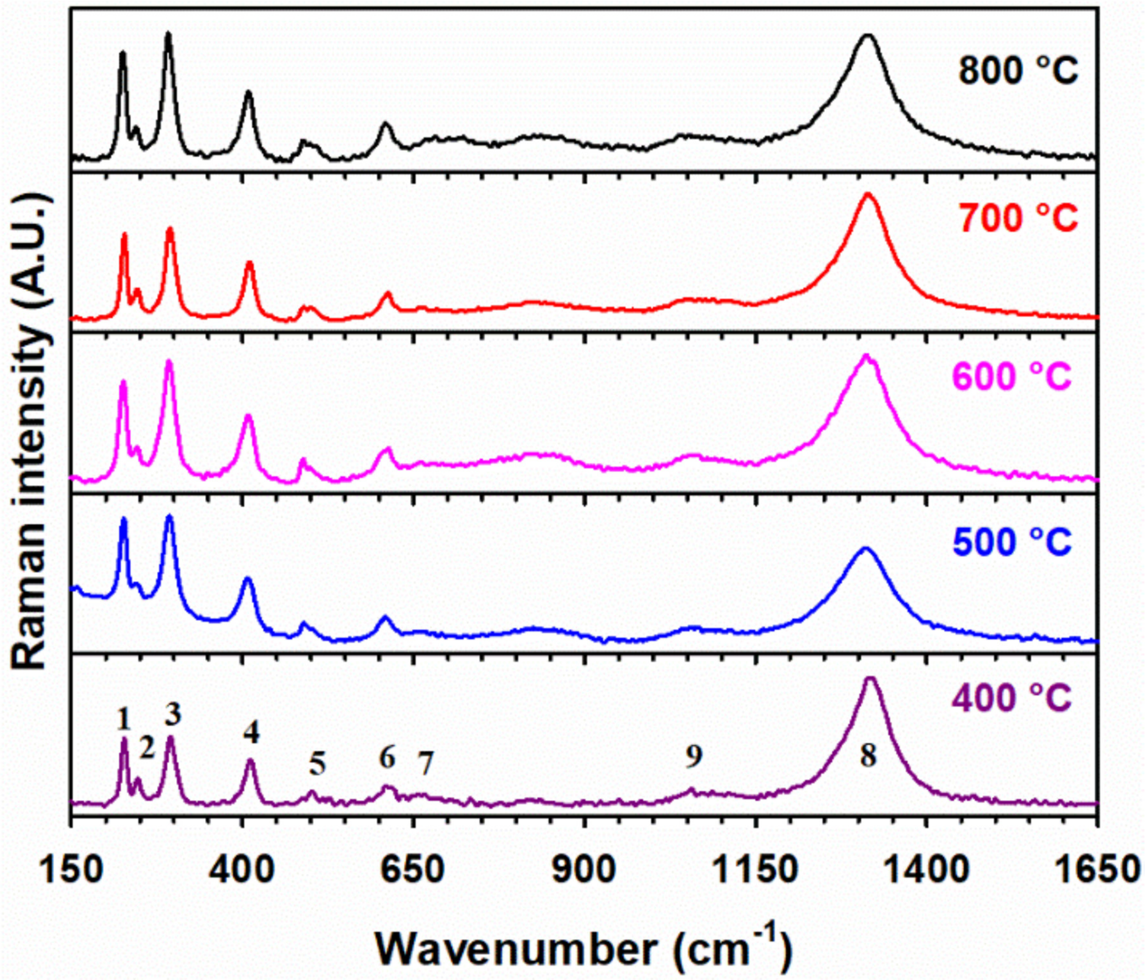

Figure S3. Raman spectra of 100 Fe:5 K for the calcination temperatures indicated in each panel. The numbers 1 to 8 correspond to modes of the hematite phase, while peak 9 relates to a mode of NO_3– in the range 980–1150 cm^{-1}.

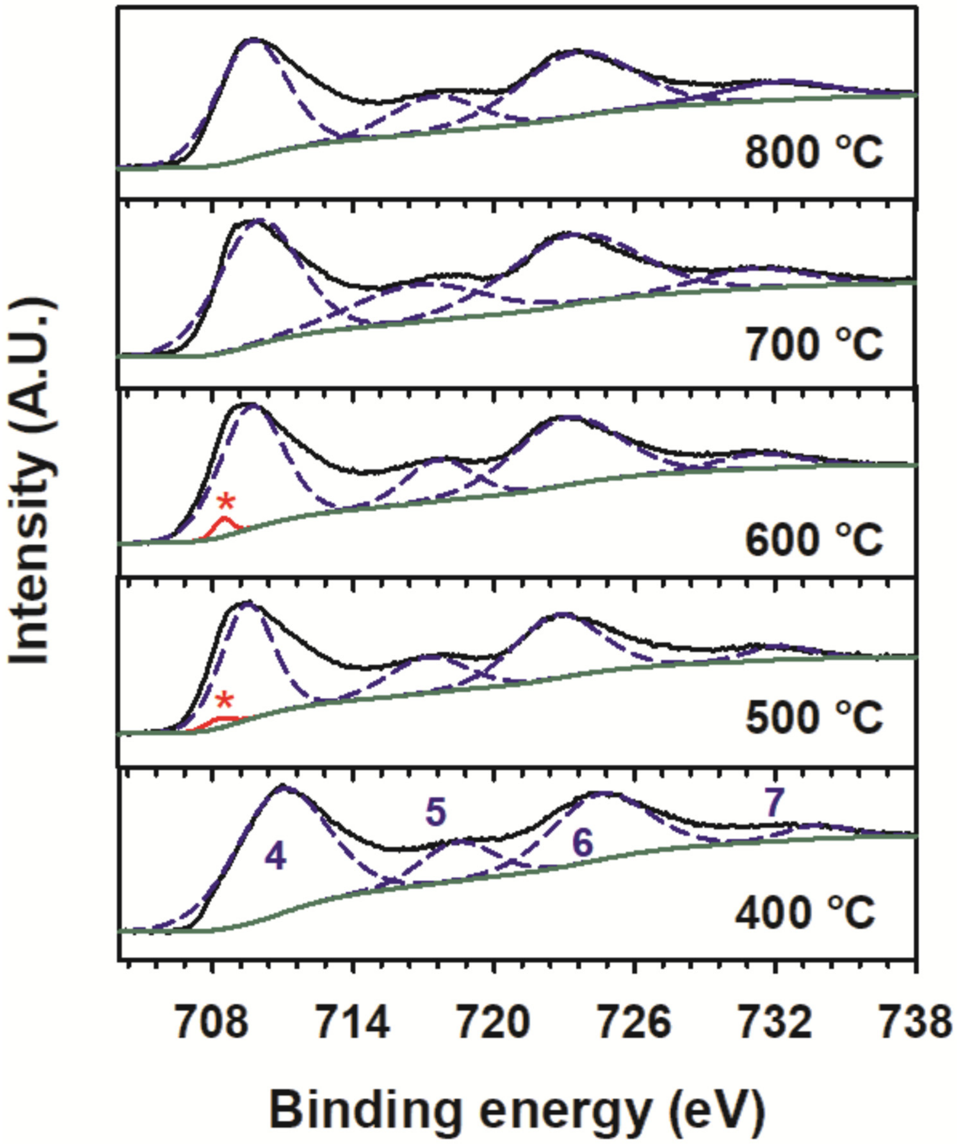

Figure S4. (Black trace) High-resolution XPS spectra of Fe(III) 2*p* for 100 Fe:5 K calcined at the indicated temperatures . (Dashed blue) Fitted peaks numbered (4) Fe(III) $2p_{3/2}$, (5) Fe $2p_{3/2}$ satellite, (6) Fe(III) $2p_{1/2}$, and (7) Fe(III) $2p_{1/2}$ satellite. (Solid green) Fitted background. (Solid red) Fitted peak marked * is assigned for Fe(II) 2*p* for the samples calcined at 500 °C and 600 °C. .

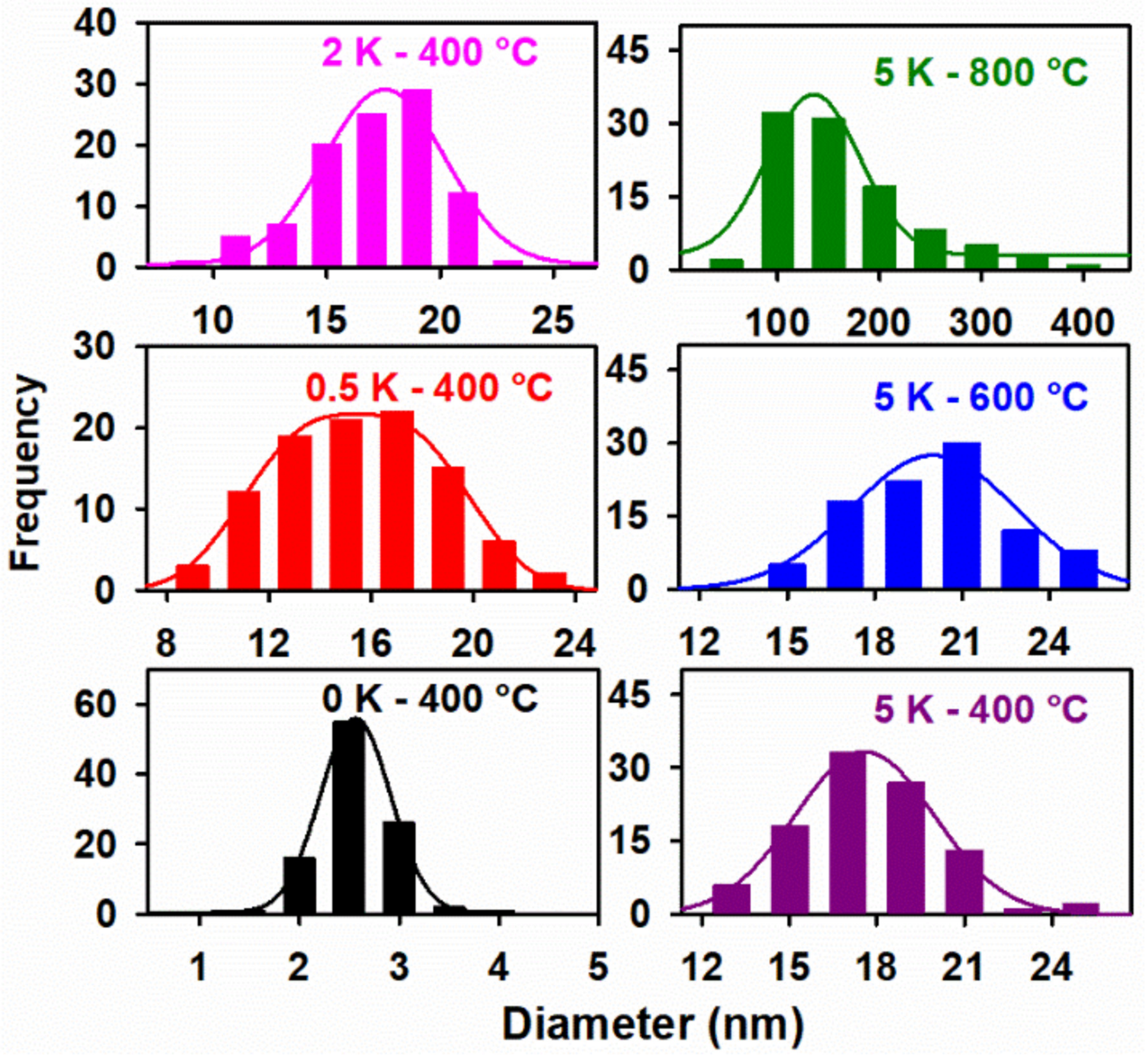

Figure S5. Particle diameter distribution histograms obtained from transmission electron micrographs of selected 100 Fe:*x* K materials as a function of potassium loading (*x*) and calcination temperature. The bar and line represent measured distribution and Gaussian fittings. .

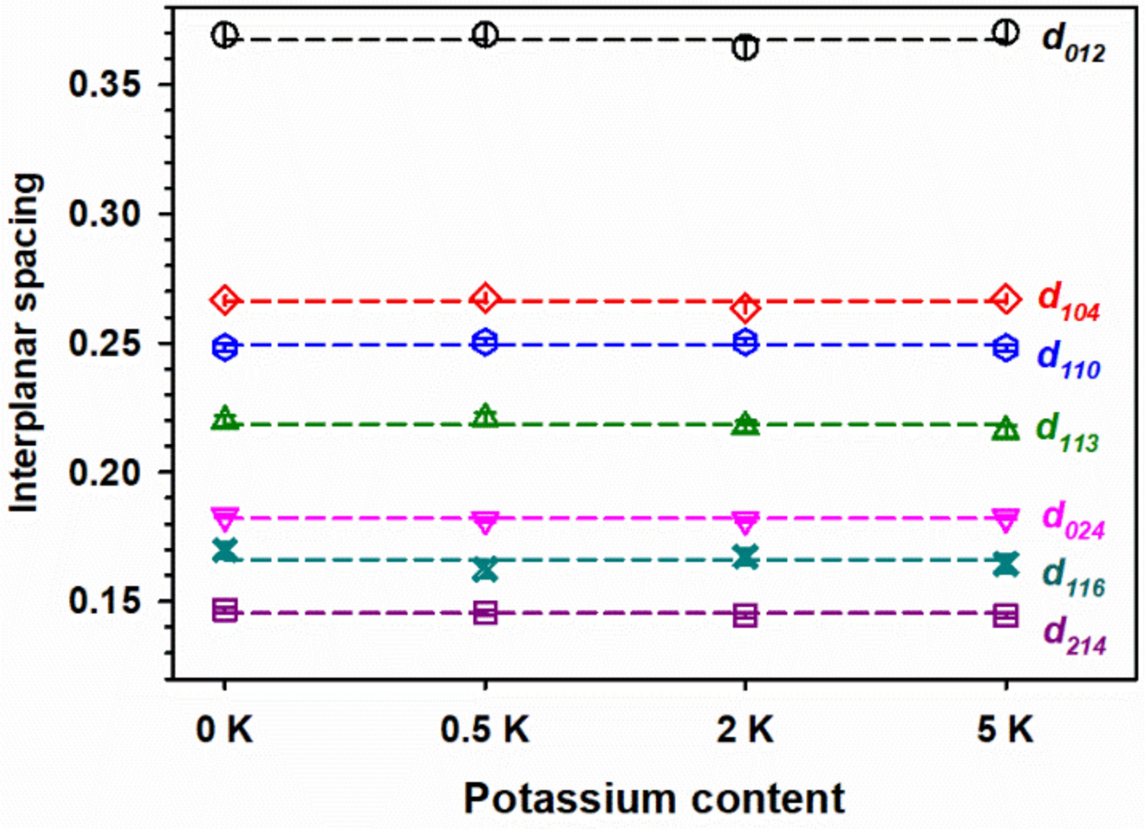

Figure S6. Interplanar spacing (d_{hkl}) from selected area electron diffraction (SAED) patterns in 100 Fe:x K materials for the indicated potassium contents (x). .

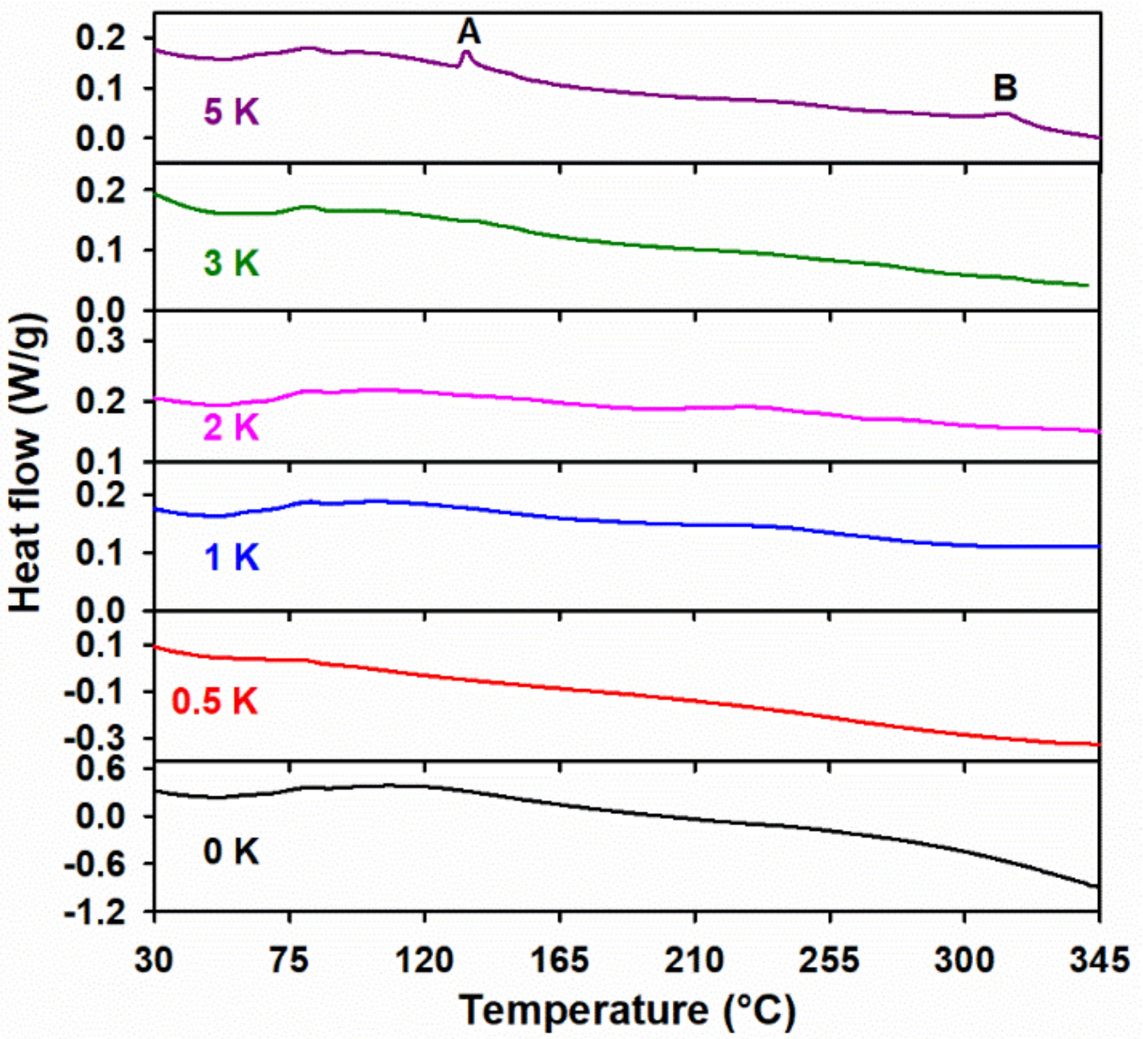

Figure S7. Differential scanning calorimetric (DSC) curve for 100 Fe:x K calcined at 400 °C with the indicated potassium contents (x). Peaks A and B correspond to the orthorhombic to rhombohedral phase transition of potassium nitrate and the melting of potassium nitrate. .

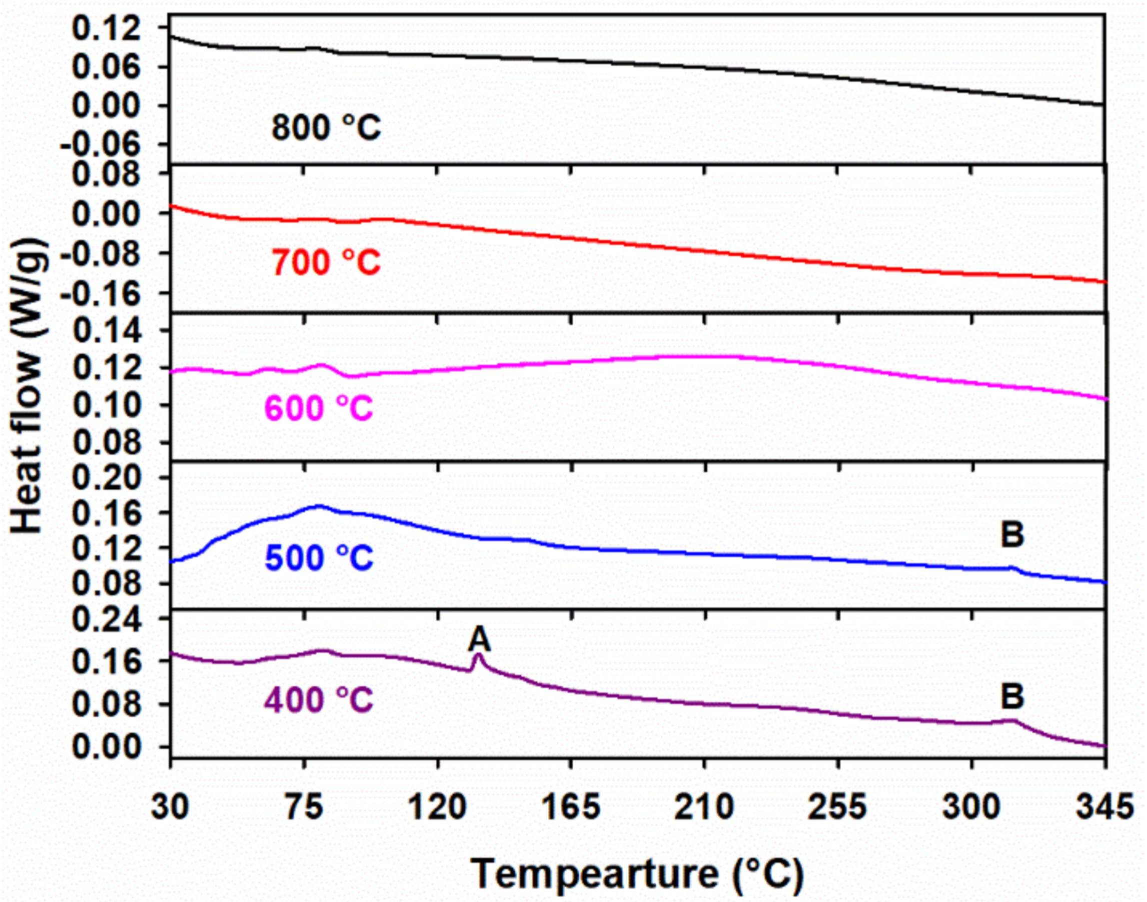

Figure S8. Differential scanning calorimetric (DSC) curve for 100 Fe:5 K at the indicated calcination temperatures. Peaks A and B correspond to the orthorhombic to rhombohedral phase transition of potassium nitrate and the melting of potassium nitrate. .

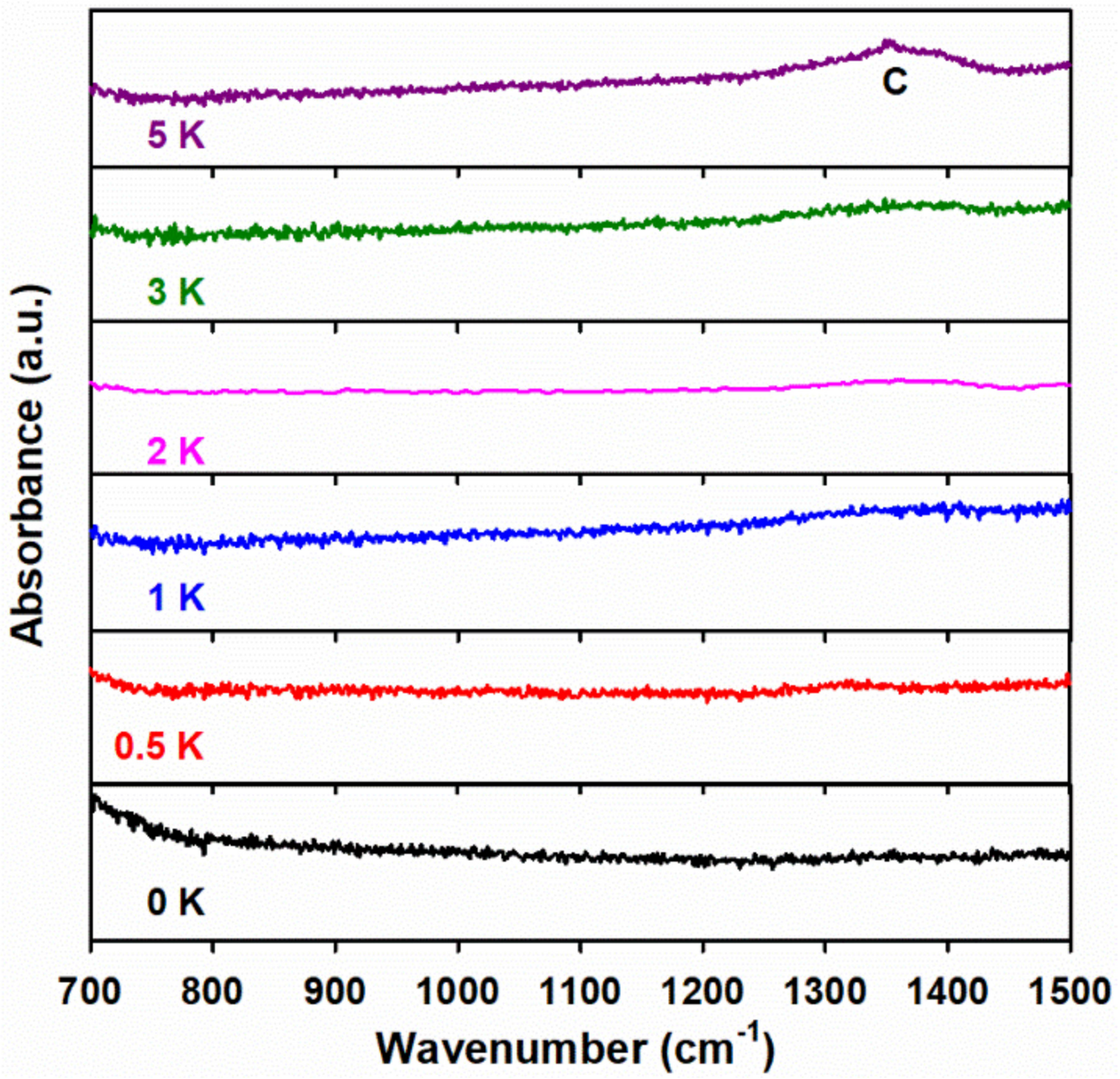

Figure S9. Fourier-transform infrared (FTIR) spectra of 100 Fe:*x* K calcined at 400 °C with the indicated potassium contents (*x*). Peak C corresponds to N–O stretching vibration in potassium nitrate.

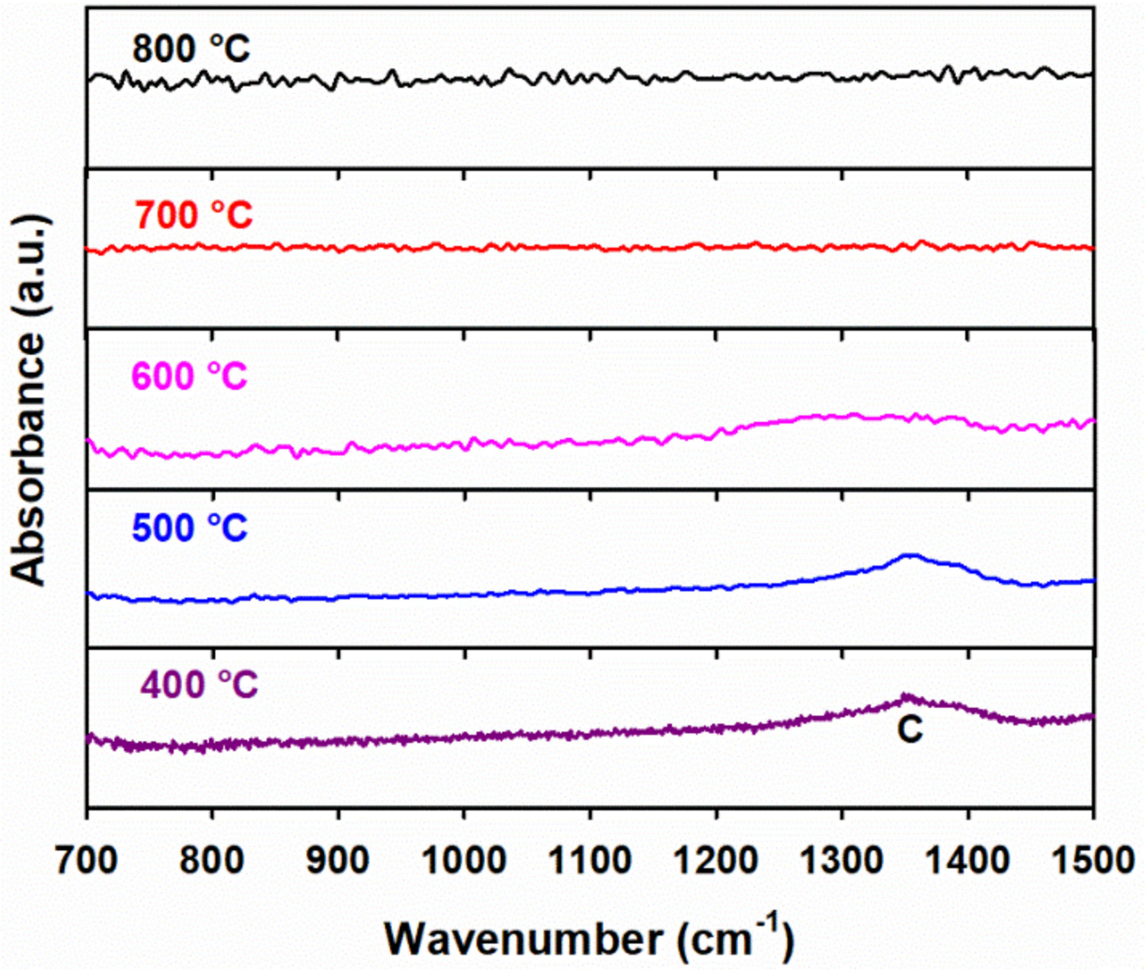

Figure S10. Fourier-transform infrared (FTIR) spectra for100 Fe:5 K at the indicated calcination temperatures. Peak C corresponds to N–O stretching vibration in potassium nitrate. .

Table S1. Surface Composition Ratio of Potassium and Iron (K/Fe) in Experiments under Variable Potassium Loading (x) and Calcination Temperature (T) .

**x** [i]	**T (°C)**	**K/Fe** [ii]
0	400	0.00
½	400	0.05
1	400	0.10
2	400	0.10
3	400	0.10
5	400	0.10
5	500	0.07
5	600	0.06
5	700	0.06
5	800	0.05

[i] Atomic ratio of potassium for 100 Fe:*x* K (0 *x* 5). [ii] Ratio of potassium to iron concentration obtained from the integration under the fitting curves of Fe 2*p*3/2 and K 2*p*3/2 in Figures 6, 7, and 8.

References

1. 1. Kramer, C.M.; Munir, Z.A.; Volponi, J.V. Differential Scanning Calorimetry of Sodium and Potassium Nitrates and Nitrites. *Thermochim. Acta* **1982**, *55*, 11-17, doi:10.1016/0040-6031(82)87002-0.

2. 2. C. Li; N. Yan; Y. Ye; Z. Lv; X. He; J. Huang; N. Zhang. Thermal analysis and stability of boron/potassium nitrate pyrotechnic composition at 180 C. *Appl. Sci.* **2019**, *9*, 3630, doi:10.3390/app9173630.
3. 3. Bykova, E.; Dubrovinsky, L.; Dubrovinskaia, N.; Bykov, M.; McCammon, C.; Ovsyannikov, S.V.; Liermann, H.P.; Kupenko, I.; Chumakov, A.I.; Rüffer, R., et al. Structural Complexity of Simple Fe2O3 at High Pressures and Temperatures. *Nat. Commun.* **2016**, *7*, 1-6, doi:10.1038/ncomms10661.
4. 4. Brooker, M.H.; Irish, D.E. Infrared and Raman Spectral Studies of KNO2–KNO3 Solutions. *Can. J. Chem.* **1968**, *46*, 229-233, doi:10.1139/v68-036.
